# Qigong-Based Therapy for Treating Adults with Major Depressive Disorder: A Meta-Analysis of Randomized Controlled Trials

**DOI:** 10.3390/ijerph16050826

**Published:** 2019-03-07

**Authors:** Lijuan Guo, Zhaowei Kong, Yanjie Zhang

**Affiliations:** 1 College of Physical Education, Shanxi Normal University, Linfen 041000, China; 20141254@sxnu.edu.cn; 2 Faculty of Education, University of Macau, Macau, China; zwkong@umac.mo; 3 Health and Exercise Science Laboratory, Institute of Sports Science, Seoul National University, Seoul 08826, Korea

**Keywords:** mind–body exercise, Qigong, Tai Chi, mental disorder, emotion dysfunction

## Abstract

This current meta-analysis review was conducted to examine the effectiveness of Qigong-based therapy on individuals with major depressive disorder. Six electronic databases (PubMed, PsycINFO, Cochrane Library, and Web of Science, Chinese National Knowledge Infrastructure, and Wangfang) were employed to retrieve potential articles that were randomized controlled trials. The synthesized effect sizes (Hedges’ g) were computerized to explore the effectiveness of Qigong-based therapy. Additionally, a moderator analysis was performed based on the control type. The pooled results indicated that Qigong-based therapy has a significant benefit on depression severity (Hedges’ g = −0.64, 95% CI −0.92 to −0.35, *p* < 0. 001, I^2^ = 41.73%). Specifically, Qigong led to significantly reduced depression as compared to the active control groups (Hedges’ g = −0.47, 95% CI −0.81 to −0.12, *p* = 0.01, I^2^ = 22.75%) and the passive control groups (Hedges’ g = −0.80, 95% CI −1.23 to −0.37, *p* < 0.01, I^2^ = 48.07%), respectively. For studies which reported categorical outcomes, Qigong intervention showed significantly improved treatment response rates (OR = 4.38, 95% CI 1.26 to 15.23, *p* = 0.02) and remission rates (OR = 8.52, 95% CI 1.91 to 37.98, *p* = 0.005) in comparison to the waitlist control group. Conclusions: Qigong-based exercises may be effective for alleviating depression symptoms in individuals with major depressive disorder. Future well-designed, randomized, controlled trials with large sample sizes are needed to confirm these findings.

## 1. Introduction

Clinical depression, also referred to as major depressive disorder (MDD), is a common debilitating mental disorder and is a leading cause of disability [1]. In fact, according to the statistics of the World Health Organization, there are more than 300 million individuals with MDD worldwide [2]. Patients with MDD often receive various forms of treatment (i.e., psychotherapy, pharmaceutics (antidepressants), or a combination of these two first-line therapies) [3]. Although these treatments have been shown to have therapeutic effects for those with MDD [4,5], they are subject to several limitations, such as high dropout rates (e.g., 12–40% during an 8-week psychotherapy), low remission rates, and numerous medication-related side effects (e.g., nausea, diarrhea, excessive sedation, and sleep problems) [6,7,8]. Considering the drawbacks of these first-line therapies, alternative and complementary therapies are needed to be developed to treat MDD [9].

Qigong is a traditional, self-healing, mind–body therapy that originated from traditional Chinese medicine (TCM), dating back to nearly four thousand years ago [10,11,12]. The term Qigong consists of two Chinese characters: Qi (vital energy) and Gong (skill acquisition through great effort) [13,14]. According to the textbook used in TCM schools in China, Qigong encompasses all mind–body exercises and skills that integrate breathing adjustment, body adjustment, and mind adjustment into one [15]. Aside from medical Qigong, Qigong is commonly categorized into two practice modalities: Movement Qigong and static Qigong [16]. Movement Qigong involves mind–body exercises that incorporate bodily movements or postures with breathing regulation and mind adjustment, such as Tai Chi (Taiji) Quan, Baduanjin, Muscle–Tendon Change Classic (Yi Jin Jing), and Five Animal Exercise (Wu Qin Xi) [17,18,19,20,21,22]. Static Qigong mostly involves meditative practices (Zen meditation or mindfulness) in either a sitting or standing posture, and does not include any bodily movement [23]. 

Qigong has gained increasing popularity across the globe and has been widely practiced by individuals with different health conditions since the development of the Chinese National Qigong Association in 2004 [24,25]. Of note, the National Health Interview Survey showed that both movement and static Qigong have been considered to be one of the most popular mindfulness-based exercises (e.g., Qigong and Yoga) in the US workforce, and more than 130 million people engaged in at least one of these exercises in the past 12 months [26]. According to the TCM principle, Qi (a subtle energy) exists in all life and circulates through multiple meridian systems in the human body, like neuropathways [27]. Subsequently, a free-flowing, well-balanced Qi system is believed to help people stay healthy, and physical or mental illnesses are said to occur as a result of a Qi blockage in some regions of the human body [27]. Therefore, Qigong, as a Chinese traditional therapy, is believed to break Qi blockages and help Qi flow smoothly, which may potentially prevent or decelerate the progression of psychosomatic illnesses [27]. The literature also suggests that Qigong is effective in improving disease-related symptoms in people with different illnesses and chronic conditions (e.g., multiple sclerosis, stroke, ankylosing spondylitis, osteoporosis, and chronic musculoskeletal pain) [28,29,30,31]. 

Although recent reviews have investigated the therapeutic effects of Qigong practice on mood regulation, such as depression and anxiety [32,33,34,35], it is unclear whether the recruited participants were clinically diagnosed with MDD. To this end, it is impossible to single out whether Qigong is effective in treating MDD based on recent reviews. The current systematic review is, therefore, conducted to critically evaluate the efficacy of Qigong-based therapy among patients who are clinically diagnosed with MDD. The results of this review shall provide evidence-based guidance for clinicians and health professionals to use or integrate Qigong therapy with other mainstream therapies for treating MDD.

## 2. Materials and Methods

This meta-analysis review was performed consistent with the Preferred Reporting Items for Systematic Review and Meta-Analysis (PRISMA) guidelines [36].

### 2.1. Information Sources and Search

The review author systematically searched six major electronic databases (PubMed, PsycINFO, Cochrane Library, Web of Science, Chinese National Knowledge Infrastructure, and Wanfang) and included no restrictions on the date of publication. The literature search was performed by entering three groups of terms: (1) Qigong/Qi Gong, Baduanjin, Tai Chi/Tai Ji, Taiji Quan, Zhuang Gong, Chi Kung, Chi therapy, Wu Qin Xi, Chan, Zen, Tai Chi, meditation, or mindfulness; (2) major depression, clinical depression, depressive disorder, or unipolar depression; and (3) randomized, randomization, or randomized controlled trial. Furthermore, we also manually searched the potentially relevant studies provided in the reference lists of relevant reviews. 

### 2.2. Eligibility Criteria and Study Selection

Inclusion criteria for identified articles were studies that: (1) were published in peer-reviewed journals; (2) used a randomized controlled design; (3) included patients aged 18 or above who had been diagnosed with MDD; (4) used any form of Qigong as a core intervention; (5) had a passive or active control group as a comparison, such as a waitlist, usual care, pharmacological therapy, or walking; and (6) reported either response rate, remission rate, and/or depression severity. The exclusion criteria were: (1) individuals diagnosed with other primary psychiatric disorders (e.g., major anxiety disorder) or mild cognitive impairment; (2) conference proceedings, case-studies, cross-sectional studies, controlled studies with no randomization or review; (3) non-Qigong interventions; and (4) duplicated publications.

Regarding study selection, removing both irrelevant and duplicate articles was conducted by an investigator screening the titles and abstracts. Two investigators then independently conducted full-text reviews to screen the remaining articles. All disagreements between the two investigators were resolved by seeking a third party’s viewpoint. 

### 2.3. Data Extraction

The following information was extracted to a data extraction table: Reference (first author and year of publication), participant characteristics (diagnostic criteria, percentage of MMD, study location, sample size, dropout rate, percentage of female participants, ethnicity, and mean age), intervention protocol (Qigong type, group or individual practice, qualification of instructor, intervention duration and follow-up period, training dosage, Qigong-related adverse events, and co-intervention), outcome measures, and study quality (allocation concealment, intention-to-treat analysis, and blinding of assessors) [37]. Quantitative data were extracted as well, including the sample size, mean and standard deviation (SD) of outcome measures at baseline and post-intervention in each group. 

### 2.4. Study Quality Assessment

Three critical items (allocation concealment, intention-to-treat analysis, and blinding of assessors) were employed to assess the methodological quality of the selected studies based on the existing literature [38,39]. Since poor randomization methods can exaggerate estimated treatment effects [40], the implementation of allocation concealment is critical. The allocation concealment is adequate if the central randomization (site remote from study location) or opaque sequentially-numbered envelopes were clearly described. A study was identified as an intention-to-treat analysis if all of the initially assigned participants in each group were included in the final data analysis, regardless of missing data or dropout during/after the intervention. Blinding means that people who performed the outcome assessments did not possess information about group allocation of the study participants. 

### 2.5. Quantitative Data Synthesis and Analysis

The Comprehensive Meta-Analysis Version 2 Software (BioStat, Englewood, NJ, USA) was employed to perform the data synthesis. The synthesized effect size (Hedges’ g), which expresses the magnitude of Qigong effects on the severity of depressive symptoms, was calculated with a 95% confidence interval (CI). The magnitude of Hedges’ g was then classified as being either a small, medium, or large effect (small effect = 0.2, medium effect = 0.5, large effect = 0.8), with a negative value indicating lower depression severity due to the beneficial effects of Qigong in comparison to the control groups. Odds ratios (OR) with 95% CIs were calculated for both response rates and remission rates. Since the measuring instruments across the selected studies were different, a random-effects model was adopted. Study heterogeneity was assessed using the value of I-squared and were divided into three levels (low = 25% or below; medium = 25% to 50%; large = 75% to 100%) [37].

There were two trials that included more than one control group [41,42]. To reduce the unit-of-analysis error, the shared Qigong group was separated into two controls for the two comparisons, while the means and SD of the Qigong group were unchanged. In addition, the Beck Depression Inventory (BDI) and the 17-item Hamilton Depression Rating Scale (HAM-D17) were used to measure the depression severity in two trials [41,42]. Given that the HAM-D17 is the clinician-administered depression assessment scale [43] and the most frequently used instrument in the present review, we only included the quantitative data measured by the HAM-D17 for meta-analysis. Furthermore, control type may be a potential moderator that also has an influence on the outcomes. Therefore, we performed two comparisons (Qigong vs. active control and Qigong vs. passive control) of the meta-analysis separately. Finally, the Egger’s test was applied to evaluate the publication bias [44].

## 3. Results

### 3.1. Study Selection and Characteristics

**Figure 1:**
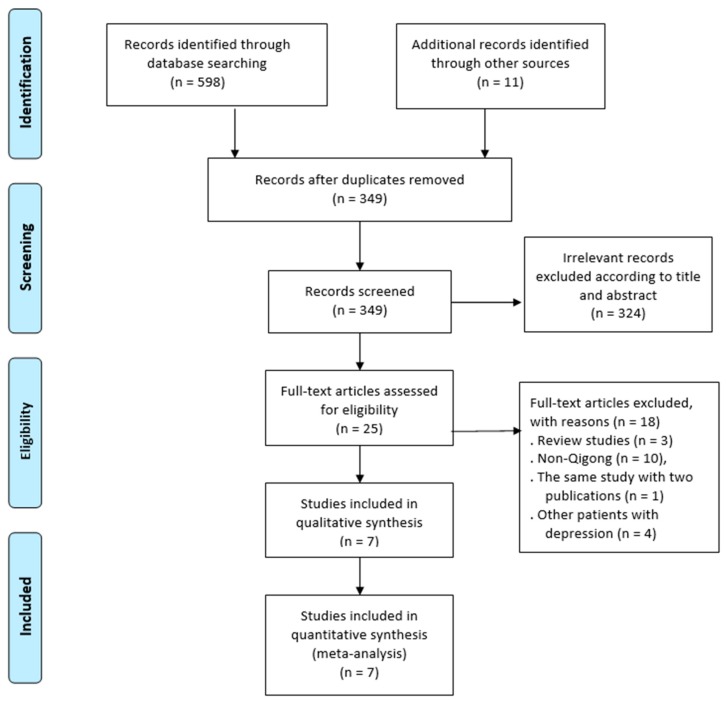
The process of study identification, screening, and selection.

Figure 1 depicts the detailed process of study selection. A total of 609 records were initially identified, seven randomized controlled trials with nine-arm were included for synthesized analysis [41,42,45,46,47,48,49]. As shown in Table 1, the seven trials were published between 2004 and 2017. Within the trials altogether, there were 382 patients with MDD in either the Qigong group (*n* = 175) or control group (*n* = 207). The holistic trials were distributed in the USA (*n* = 3), China (*n* = 2), United Kingdom (*n* = 1), and Germany (*n* = 1). The Qigong therapy lasted from 1 to 12 weeks, but only one study used a 12-week follow-up assessment [41]. A group-based training mode was the most frequently used method in the Qigong practice (antidepressant medications and/or psychotherapy allowed), without adverse events reported. Depression severity was measured by four difference scales (HAM-D17, HAM-D24, BDI, and the Epidemiological Studies Depression Scale). Response rate (categorized as >50% improvement on the HAM-D17 score) and remission rate (HAM-D17 scores ≤ 7) were reported in two trials [41,45].

### 3.2. Quality Assessment

Only two studies met all the three of critical criteria, defined as allocation concealment, intention-to-treat analysis, and blinding of assessors [45,46]. Allocation was adequately concealed in five trials [42,45,46,48,49], four studies [41,45,46,47] were not used intention-to-treat analysis, and assessors were blinded to group assignment in four trials [42,45,46,47].

### 3.3. Effects of Qigong on Response Rate, Remission Rate, and Depression Severity

There were only two trials reporting response rate and remission rate. Of the two trials, the study by Yeung et al. [45] included a Qigong group and a passive control group, while two control groups (active and passive conditions) were used in the other study [42]. An odds ratio was computed across these two studies and the results showed that the Qigong intervention showed significant improvement in treatment response rate (OR = 4.38, 95% CI 1.26 to 15.23, *p* = 0.02) and remission rate (OR = 8.52, 95% CI 1.91 to 37.98, *p* = 0.005) compared with the waitlist control group. There was no significant publication bias for the seven included studies with the nine treatment arms based on the results of Egger’s test (*β*= −1.25, *p* = 0.51). 

**Figure 2:**
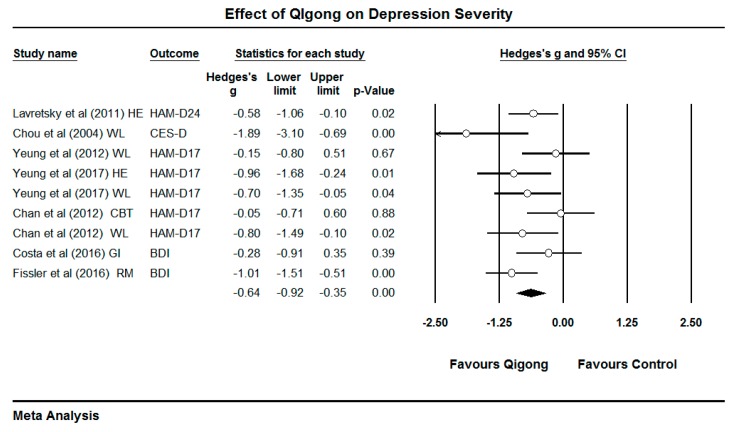
Effects of Qigong on depression severity. Note: HAM-D24 = 24-item Hamilton Depression Rating Scale, HAM-D17 = 17-item Hamilton Depression Rating Scale, CES-D = Epidemiological Studies Depression Scale, BDI = Beck Depression Inventory. HE = heath education, WL = waitlist, CBT = cognitive behavioral therapy, GI = guided imagery, RM = rest and music.

In term of depression severity, the synthesized result indicated that Qigong therapy contributed to a more significant benefit than the control group (Hedges’ g = −0.64, 95% CI −0.92 to −0.35, *p* < 0. 001, I^2^ = 41.73%). Moreover, the effects of Qigong significantly reduced depression as compared to the active control group (Hedges’ g = −0.47, 95% CI −0.81 to −0.12, *p* = 0.01, I^2^ = 22.75%) and passive control group (Hedges’ g = −0.80, 95% CI −1.23 to −0.37, *p* < 0.01, I^2^ = 48.07%), respectively (Table 2). Interestingly, there were two types of Qigong therapy (movement Qigong and static Qigong). The movement Qigong from seven sets of data had the benefit of significantly reducing depression (Hedges’ g = −0.62, 95% CI −0.96 to −0.28, *p* < 0.01, I^2^ = 41.62%). The static Qigong showed a marginal significant difference in reducing depression compared with control group (Hedges’ g = −0.67, 95% CI −1.38 to 0.04, *p* = 0.06, I^2^ = 67.84%). However, no statistically significant difference between movement Qigong and static Qigong on reducing depression was observed (*p* = 0.90) (Table 2). Additionally, according to Table 1 and Figure 2, the long Qigong practice time tends to have great benefits in movement Qigong [45,47], and the corresponding training protocols were each exercise session (45–60 min), frequency (2–3 times/week), and exercise duration (12 weeks). In static Qigong (mindfulness) intervention, it indicated that the protocol was fine, if participants persisted for 50 min once a day for two weeks.

## 4. Discussion

This meta-analytic review was conducted to statistically synthesize the evidence of the effectiveness of Qigong-based therapy on MDD. The findings from our review suggest that the group of TCM mind-body exercise shows some clinical effect in the treatment of MDD as compared to active/passive controls.

In terms of response and remission rates, more than 50% improvement on the HAM-D17 scores and HAM-D17 scores ≤ 7 were identified as positive effect respectively [45]. Results from this meta-analysis indicated that Qigong-based intervention may be significantly beneficial to improve the response and remission rate for MDD compared with the control group (*p* < 0.05). These results were in line with previous clinical study [41], which agreed that the participants who received 60 min of Tai Chi twice a week for 12 weeks achieved some improvement in their depression than other therapies (i.e., psychoeducation). It is likely that Qigong-based intervention combining intentional bodily movements, rhythmic respiration and mind adjustment can regulate the body’s stress and then reduce depression symptoms [9]. Therefore, depression severity was significantly alleviated in our meta-analytic study. In addition, according to the different Qigong types, movement Qigong had a significant improvement in alleviating the depression (*p* < 0.01), and there was a marginal significant improvement on depression through practicing static Qigong (*p* = 0.06). However, the moderate effect sizes of alleviated depression symptoms were tentative because of including a small number of eligible studies in this current review. Further studies were needed to examine the efficacy of either movement Qigong or static Qigong or both Qigong styles on treating MDD. 

Although Qigong-based intervention has a potential impact on clinical and public health, the exact mechanisms of how Qigong works in reducing MDD are unknown, using Qigong as an important adjunct therapy for treatment of MDD was positively effective based on the findings from this study. A contemporary concept hypothesizes that Qigong practitioners are able to combine their awareness of spirit with body posture, breathing, and activity to elevate physiological proprioception. Furthermore, it can enhance homeostasis of the internal environment [50]. There are a lot of advantages of using Qigong exercises, compared to the current first-line treatments (drugs and cognitive behavioral therapy), as an alternative method for treating individuals with MDD. Qigong exercises are characterized as easy to learn, and have few side effects, and are popular in a number of people across all ages and physical condition. In particular, Qigong exercise is an additional option for older adults with medication side effects. However, there are some potential disadvantages in actual life, such as a lack of qualified Qigong instructors in some places, non-standardized Qigong exercises, and non-adherence to long-term practices.

Several methodological limitations need to be noted. First, the subjective bias may exist in participants. Although eligible randomized controlled trials were included, participants still knew the allocation of the intervention (i.e., if they received Qigong training or not). Second, in most studies, participants were given mixed treatment, such as a combination of medication and Qigong. To some extent, it is difficult to determine if the Qigong alone, both Qigong and medication (synergetic intervention effect), or the usual treatment can contribute to alleviating their symptoms. However, significant effects of Qigong, as a complementary therapy, were observed to treat depressive disorder in this current study. Third, as large spans of time of Qigong practice were employed in the included studies, an optimal Qigong prescription was not easy to recommend for patients. It is possible that Qigong provides a number of benefits for human health based on practitioners daily self-practice, such as movement Qigong (45–60 min, 2–3 times/week) and static Qigong (50 min, once a day). Lastly, it is an interest point that, as both types of Qigong were effective through integrating a small number of studies, future studies may investigate whether the combination of movement Qigong and static Qigong are effective for MDD.

## 5. Conclusions

Our meta-analysis results suggest that Qigong may be an effective complementary intervention to treat individuals with MDD. Participants may practice movement Qigong for 45–60 min, two to three times weekly, for 12 weeks, and practice static Qigong for 50 min every day in a clinic. As there are some methodological limitations in the empirical studies included, carefully interpreting these findings is required. Meanwhile, to further investigate the efficacy of Qigong-based intervention on MDD, it is necessary to study the quality of randomized controlled trails with well-researched design.

## Figures and Tables

**Table 1:** Characteristics of the eligible randomized controlled trials.

Reference	Characteristics of Participants			Intervention Protocol				Outcome Measured	Study Quality		
	Patients Diagnostic Criteria, % of MDD, Study Location	Total Sample Size (DR), Female (%), Predominant Ethnicity	Age: Mean (SD)	Training Prescription	Total Training Min	Grp or Ind	Co-intervention	Outcomes (Instrument)	Concealed Allocation	Intention to Treat Analysis	Blinded
Yeung et al. (2017) [41]	DSM-IV (psychiatrist), 100% of MDD, USA	67 (25.3%), 72% female, 100% Chinese Americans	54 (13)	Q: 2 × 60 min/week, 12 weeks (12 weeks follow-up) (Tai Chi); C1: 2 × 60 min/week (health education); C2: wait-list	1440	Grp	None	Remission rate, response rate, depression severity (HAM-D17 and BDI)	No	Yes	No
Chan et al. (2012) [42]	DSM-IV-TR (psychiatrist), 100% of MDD, China (HK)	75 (33.3%), 80% female, 100% Chinese	Q: 47.1 (9.5) C1: 46.9 (6.5) C2: 45.4 (8.3)	Q:1 × 90 min/week 10 weeks (Shaolin movement Qigong); C1: 1 x× 90 min/week (CBT); C2: wait-list	900	Grp	Antidepressants	Depression severity (HAM-D_17_ and BDI)	Yes	No	Yes
Yeung et al. (2012) [45]	DSM-IV (psychiatrist), 100% of MDD, USA	39 (5.1%), 77% female, 100% Chinese Americans	55(10)	Q: 2 × 60 min/week, 12 weeks (Tai Chi); C: wait-list	1440	Grp	Antidepressants allowed	Remission rate, response rate, depression severity (HAM-D17)	Yes	Yes	Yes
Lavretsky et al. (2011) [46]	DSM-IV (psychiatrist), 100% of MDD, USA	73 (6.8%), 61.6% female, 74% Caucasians and 11% African-Americans	Q: 69.1 (7.0) C: 72.0 (7.4)	Q: 1 × 120 min/week, 10 weeks (Tai Chi); C: 1 × 120 min/week (health education)	1200	Grp	Escitalopram/lorazepam: 10–20 mg/day	Depression severity (HAM-D24)	Yes	Yes	Yes
Chou et al. (2004) [47]	DSM-IV (psychiatrist), 90% of MDD, China (HK)	14 (0%), 50% female, 100% Chinese	72.6 (4.2)	Q: 3 × 45 min/week, 12 weeks (Tai Chi); C: wait-list	1620	Grp	Antidepressants or psychotherapy	Depression severity (CES-D)	No	Yes	Yes
Costa et al. (2016) [48]	DSM-IV (psychiatrist), 100% of MDD, UK	40 (7.5%), 74% female, 74% Caucasians and 16% African-Americans	Q: 39 (1.2) C: 38 (9.7)	Q: 1 × 60min/week (Grp) + 6 × 30 min/week (Ind), 1 week (static Qigong); C: same dosage/format (guided imagery)	240	Mixed	Antidepressants	Depression severity (BDI)	Yes	No	No
Fissler et al. (2016) [49]	DSM-IV (a psychiatrist), 100% of MDD, Germany	74 (8.1%), 56% female, 100% Caucasians	42 (12.5)	Q: 7 × 50 min/week, 2 weeks (Static Qigong); C: 7 × 50 min/week (rest and music)	700	Grp	Antidepressants	Depression severity (BDI)	Yes	No	No

Note: MDD = major depressive disorder; DR = dropout rate; FU = follow-up period; AE = adverse event; SD = standard deviation; Q = Qigong group; C = control group; HK = Hong Kong; DSM-IV = Diagnostic and Statistical Manual of Mental Disorders; Grp = group based practice; Ind = individual practice; CES-D = Epidemiological Studies Depression Scale; HAM-D_17_ = 17-item Hamilton Depression Rating Scale; BDI = Beck Depression Inventory; HAM-D_24_ = 24-Item Hamilton Depression Rating Scale; Remission rate = HAM-D17 scores ≤ 7; Response rate = ≥ 50% improvement on the HAM-D17 score; CBT = cognitive behavioral therapy.

**Table 2:** Moderator analysis for Qigong versus control group.

Categorical Moderator	Outcome	Level	No. of Studies/ Comparisons	Hedges’ g	95% CI	I^2^ %	Test for Between-Group Homogeneity		
							*Q*-Value	df (*Q*)	*p*-Value
Control Type	Depression	Active	4	−0.47	−0.81 to −0.12	22.75%	1.41	1	0.23
		Passive	5	−0.80	−1.23 to −0.37	48.07%			
Qigong Type	Depression	Movement	7	−0.62	−0.96 to −0.28	41.62%	0.02	1	0.9
		Static	2	−0.67	−1.38 to 0.04	67.84%			
